# Improving vasopressor use in cardiac arrest

**DOI:** 10.1186/s13054-023-04301-3

**Published:** 2023-03-02

**Authors:** Gavin D. Perkins, Keith Couper

**Affiliations:** Warwick Clinical Trials Unit, Warwick Medical School, University of Warwick, Coventry, CV4 7AL UK

**Keywords:** Adrenaline, Advanced life support drugs, Cardiac arrest, Vasopressors, Vasopressin

## Abstract

The Chain of Survival highlights the effectiveness of early recognition of cardiac arrest and call for help, early cardiopulmonary resuscitation and early defibrillation. Most patients, however, remain in cardiac arrest despite these interventions. Drug treatments, particularly the use of vasopressors, have been included in resuscitation algorithms since their inception. This narrative review describes the current evidence base for vasopressors and reports that adrenaline (1 mg) is highly effective at achieving return of spontaneous circulation (number needed to treat 4) but is less effective on long-term outcomes (survival to 30 days, number needed to treat 111) with uncertain effects on survival with a favourable neurological outcome. Randomised trials evaluating vasopressin, either as an alternative to or in addition to adrenaline, and high-dose adrenaline have failed to find evidence of improved long-term outcomes. There is a need for future trials to evaluate the interaction between steroids and vasopressin. Evidence for other vasopressors (e.g. noradrenaline, phenylephedrine) is insufficient to support or refute their use. The use of intravenous calcium chloride as a routine intervention in out of hospital cardiac arrest is not associated with benefit and may cause harm. The optimal route for vascular access between peripheral intravenous versus intraosseous routes is currently the subject of two large randomised trials. Intracardiac, endobronchial, and intramuscular routes are not recommended. Central venous administration should be limited to patients where an existing central venous catheter is in situ and patent.

## Background

Cardiac arrest is characterised by the sudden and catastrophic loss of cardiac output [1]. The condition affects hundreds of thousands of people around the world each year in both the pre-hospital and hospital setting [2]. Unless resuscitation is started promptly and return of spontaneous circulation (ROSC) achieved rapidly, few patients survive to make a meaningful recovery [3]. Whilst the early parts of the Chain of Survival (early recognition and call for help, early cardiopulmonary resuscitation (CPR) and early defibrillation) are the most effective at improving outcomes from cardiac arrest, many patients remain refractory to these interventions [4]. Vasopressors have been used since the inception of modern-day CPR in patients where initial resuscitation efforts have failed to achieve ROSC. Early clinical guidelines produced by Safar et al. in the 1960’s recommended the inclusion of vasopressors including adrenaline, noradrenaline, phenylephrine, metaraminol and calcium in the Physicians Emergency Drug Bag [5]. This narrative review provides a state-of-the-art summary of recent evidence for vasopressor use during cardiac arrest. The reader is directed to the European Resuscitation Council and European Society Intensive Care Medicine guidelines for a detailed summary and recommendations regarding their use following ROSC [6].

## Adrenaline

The potent vasopressor effects of adrenaline have been recognised for nearly 150 years. Acting via alpha receptors located on vascular smooth muscle cells, adrenaline causes vasoconstriction, increasing aortic diastolic pressure and coronary perfusion pressure, thereby increasing the chances of ROSC. Whilst consistent and compelling evidence from animal and observational studies support a potent effect on ROSC, there was less certainty about adrenaline’s effect on patient-centred outcomes, such as 30-day survival, favourable neurological outcome, and health-related quality of life. The potentially adverse effects of adrenaline on long-term outcomes have been ascribed to increased cardiac instability after ROSC, immunomodulatory changes and reduced cerebral microvascular blood flow [7].

### Standard dose adrenaline (1 mg)

Jacobs et al. led the first randomised trial comparing adrenaline to placebo in out of hospital cardiac arrest in Western Australia (The Pre-hospital Adrenaline for Cardiac Arrest (PACA trial)) [8]. The trial set out to recruit 5000 participants but closed to recruitment after 601 participants were randomised as plans to roll out the trial across Australia and New Zealand were unsuccessful. Approximately 10% (67) participants were excluded after randomisation as information about treatment allocation was unavailable. The trial found that compared with placebo, adrenaline use significantly improved the rate of pre-hospital ROSC (8.4% versus 23.5%, odds ratio (OR) 3.4 (95% confidence interval (CI) 2.0–6.5) and hospital admission (13% versus 25.4%, OR 2.3 (95% CI 1.4–3.6). However, these early benefits did not translate into improved long-term survival or survival with a favourable neurological outcome.

Although not directly comparing adrenaline with placebo, a Norwegian study randomised 916 adults with out of hospital cardiac arrest to a strategy of drug use with a strategy of no drug use [9]. Most participants (79%) in the drug use arm received adrenaline compared to a minority (9%) in the no drug use arm. The pattern of findings was similar to the PACA trial in so far as showing a significant increase in ROSC with drug use (25% versus 40%, *P* < 0.01) and admission to hospital (29% versus 43%, *P* < 0.01) but no difference in survival to discharge or favourable neurological outcomes. An observational study which used these trial data to compare participants who actually received adrenaline with those that did not receive adrenaline found that adrenaline was associated with improved ROSC, but reduced survival to hospital discharge and worse neurological outcomes [10]. This study serves to highlight the limitations of observational studies and the influence of resuscitation time, something discussed in more detail below.

**Fig. 1:**
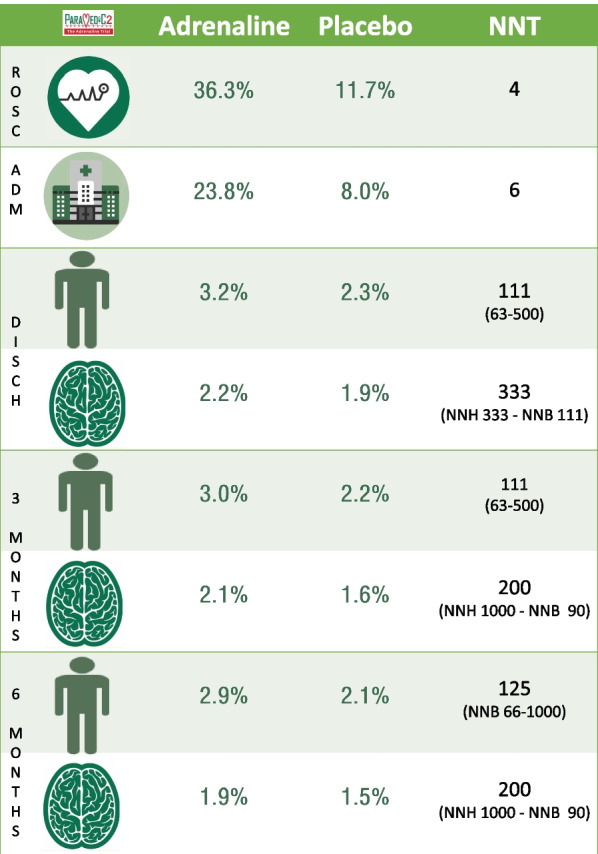
Key clinical outcomes from the PARAMEDIC2 trial. Abbreviations: NNT = number needed to treat; NNH = number needed to harm; NNB = number needed to benefit; ROSC = Return of Spontaneous Circulation, ADM = Admitted to hospital (survived event); DISCH = Discharge. Stickman picture = survival outcome; Brain picture = favourable neurological outcome

The Pre-hospital Assessment of the Role of Adrenaline Measuring the Effectiveness of Drug Administration In Cardiac arrest 2 (PARAMEDIC2) trial is the largest cardiac arrest drug trial to have been conducted to date [11–13]. The study took place in 5 National Health Service Ambulance Services in the United Kingdom. The trial enroled 8014 participants between December 2014 and October 2017. The study showed similar outcomes for ROSC and survival to admission (see Fig. 1) as to those seen in PACA and the Norwegian study. The PARAMEDIC2 study showed for the first time that adrenaline improved long-term survival, but this did not translate into improved survival with a favourable neurological outcome. The number needed to treat (NNT) summaries presented in Fig. 1 highlight the significant impact that adrenaline has on improving early outcomes (NNT 4–6), but a much smaller effect on longer term outcomes (NNT > 100).

The trial was supported by a cost effectiveness analysis assessed within-trial (6 months) and extrapolated to a lifetime horizon [14]. Mean costs were significantly higher in the adrenaline arm with only marginally higher mean quality-adjusted life years. The incremental cost effectiveness ratio was €1,946,953 (within trial) and €93,231 (lifetime horizon) per quality-adjusted life year, which falls outside the threshold typically regarded as cost effective by the UK National Institute for Health and Care Excellence.

The study found that more participants in the adrenaline arm were able to gift their organs for transplantation. In the adrenaline group, 40 donors gifted 115 organs, compared with 24 donors gifting 74 organs in the placebo group. When the decision-analytic modelling considered both the direct economic effects over the lifetimes of survivors and indirect economic effects in organ recipients, the mean incremental costs effectiveness ratio was €18,499 per quality-adjusted life year, a value likely to be considered as cost-effective in a UK setting.

### Shockable versus non-shockable rhythms

A meta-analysis of the PACA and PARAMEDIC2 trials explored the effectiveness of adrenaline according to whether the initial rhythm was shockable or non-shockable. The effect on ROSC and survival to hospital discharge was much more pronounced in those with non-shockable rhythms (ROSC non-shockable OR 6.14 (95% CI 5.28–7.15) compared to shockable OR 2.30 (95% CI 1.88 -2.82); survival to discharge non-shockable OR 2.57 (95% CI 1.36–4.83) compared to shockable OR 1.26 (95% CI 0.93–1.71) [15].

An observational study drawn from data collected as part of the US Get with the Guidelines Resuscitation Registry explored survival outcomes in 34,820 patients with in-hospital cardiac arrest due to an initially shockable rhythm. The study found that, in contrast to international guidelines, adrenaline was used before defibrillation in 9,630 (27.7%) patients. Treatment with adrenaline prior to defibrillation was strongly associated with delayed defibrillation (3 min versus 0 min). Using time-dependant propensity score matching across 9,011 matched pairs, the study found that adrenaline use before defibrillation was associated with a lower chance of survival (25.2% v 29.9%; adjusted OR 0.81 (95% CI 0.74 to 0.88) and favourable neurological survival (18.6% v 21.4%; adjusted OR 0.85 (95% CI 0.76–0.92)) [16].

### Timing of drug administration

Cardiac arrest is one of the most time critical medical emergencies. The Chain of Survival highlights the importance of early treatments through each link in the chain. Just as evidence exists for the importance of minimising the time to cardiac arrest recognition, activation of emergency services, initiation of CPR and defibrillation [17, 18], evidence exists highlighting the importance of early drug administration, particularly in those with initially non-shockable rhythms.

In an observational cohort study using data from the Get with the Guidelines Resuscitation Registry, Donnino et al.explored the influence of the time of adrenaline administration in 25,095 patients with an in-hospital cardiac arrest and an initial non-shockable rhythm [19]. The study reported a stepwise decrease in the primary outcome of survival in association with delayed adrenaline administration. Compared to the reference group of 1–3 min, the adjusted odds ratio for survival to hospital discharge when drugs were administered at 4–6 min was 0.91 (95% CI 0.82–1.00); at 7–9 min was 0.74 (95% CI 0.63–0.88) and at more than 9 min was 0.63 (0.52–0.76). A similar pattern of findings was observed for ROSC, survival to 24 h and survival with favourable neurological outcome. The findings were consistent across a series of sensitivity analyses which sought to adjust for confounding caused by delays in initiating resuscitation.

**Fig. 2:**
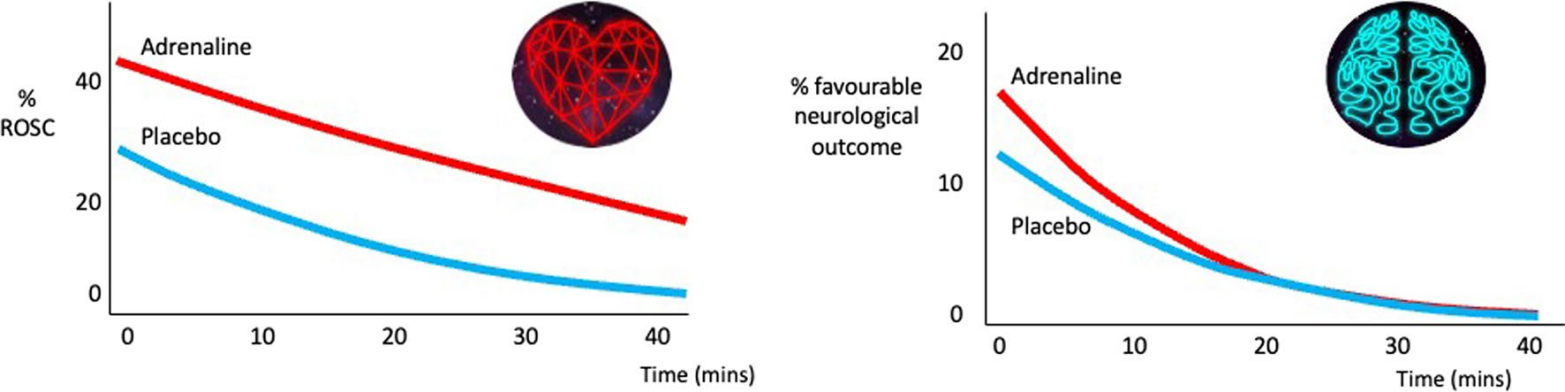
Effect of time of drug administration on return of spontaneous circulation and favourable neurological outcome. Figure is based on data presented in Perkins et al. The influence of time to adrenaline administration in the Paramedic 2 randomised controlled trial. *Intensive Care Med* 2020; 46(3): 426–36 [21]

Findings from observational studies need to be interpreted with caution due to the risk of confounding. A particular issue is that of resuscitation time bias, whereby interventions given later in a cardiac arrest (e.g. drugs, tracheal intubation) are seemingly associated with a poorer outcome. [20] Rather than being a causal relationship, the association is caused by the fact that the duration of cardiac arrest itself is associated with worse outcomes. The post hoc analysis by the PARAMEDIC2 investigators provides unique insights into the time to treatment as it was able to compare the timing of drug administration between adrenaline and placebo groups [21]. As shown in Fig. 2, the effectiveness of adrenaline relative to placebo for the outcome of ROSC remains relatively constant over time. By contrast the difference in treatment effect between adrenaline and placebo decreases over time, such that by approximately 20 min the curves converge. These findings likely reflect the greater resilience to ischaemia of the heart relative to the brain. It may also explain, at least in part, the apparent paradox of the large effect of adrenaline on ROSC, small effect on long-term survival and uncertain effect on favourable neurological outcome.

### High-dose adrenaline (5–10 mg)

The Cochrane systematic review and meta-analysis of vasopressors for cardiac arrest identified 13 (10 adult, 3 paediatric) randomised trials that compared standard dose (1 mg) with high dose (5-10 mg) adrenaline [7]. Many of the studies were conducted more than 20 years ago. Overall the certainty of evidence was rated as low or very low due to high risk of bias, inconsistency and imprecision. Meta-analysis of 13 studies (7014 participants) showed a small increase in the rate of ROSC (risk ratio (RR) 1.15 (95% CI 1.02–1.29)). However there was no difference in survival to hospital discharge (RR 1.10 (95% CI 0.75–1.62); 10 studies, 6274 participants) or favourable neurological outcome (RR 0.91 (95% CI 0.65–1.26); 4 studies, 5803 participants).

### Route of administration

A variety of routes for drug administration have been used over the years. These include intra-cardiac, central intravenous (IV), endobronchial, peripheral IV and intraosseous access (IO). Current guidelines do not recommend intracardiac or endobronchial administration due to the risk of injury / misplacement (intracardiac) or variable / uncertain absorption (endobronchial) [22, 23]. If central venous access is already in place then it should be the preferred route for drug administration due to the short transit time of drugs injected to reach the central circulation. The risks of misplacement and damage to adjacent structure through attempting central venous cannulation during CPR likely outweigh the benefits. [22, 23] The intramuscular route for adrenaline administration in cardiac arrest might facilitate very early drug administration, but ongoing uncertainties regarding its absorption preclude its routine use outside the research setting [24, 25].

The use of IO drug administration has been growing in recent years. In the PARAMEDIC2[26] one third of participants received drug treatment through the IO route whilst in the COCA (calcium for out of hospital cardiac arrest)[27] trial it was as high as two thirds. Potential advantages of the IO route are that it is easier and quicker to obtain IO rather than IV access [28]. Disadvantages include misplacement and variable drug absorption, although a post hoc analysis of survivors in the COCA trial showed similar ionised calcium levels on hospital admission between patients administered calcium chloride via the IO and IV route [29].

Observational studies comparing IO and IV drug administration are often limited by the risk of confounding due to resuscitation time bias as IO is often used as a rescue therapy after initial attempts at IV access have failed [30, 31]. The PARAMEDIC2 study, was able to provide unique insights in the potential efficacy of the IO route as it included matched patients who received placebo through both the intravenous and IO routes [26]. The study reported that the adjusted odds ratios for ROSC were similar in the IV (adjusted OR 4.07 (95% CI 3.42–4.85)) and IO groups (adjusted OR 3.98 (95% CI 2.86–5.53), p-value for interaction 0.90). Survival rates were not statistically different for long-term survival and favourable neurological outcomes between the IO and IV group.

The potential benefit of an IO first strategy in out of hospital cardiac arrest is being tested in ongoing trials in Denmark (NCT05205031)[32] and the United Kingdom (ISCRTN 14223494) [33].

### Lower doses of adrenaline or continuous infusions

Using lower doses of adrenaline or adrenaline as a continuous infusion may reduce the risks of arrythmia and other adverse effects of adrenaline. However such an approach has not yet been test in a randomised controlled trial. The EpiDOSE study (NCT03826524) is exploring lower cumulative doses of adrenaline in patients with out of hospital cardiac arrest due to an initially shockable rhythm and may provide some insights into this question.

### Dosing based on physiological response rather than fixed dosing

Paradis and colleagues were the first to report over 30 years ago on the importance of coronary perfusion pressure as a critical requirement for achieving ROSC [34]. Coronary perfusion pressure was calculated by measuring aortic pressure and subtracting right atrial pressure in 100 patients who were receiving treatment for out of hospital or emergency department cardiac arrest. The study identified a coronary perfusion pressure of > 15 mm Hg was a prerequisite to achieving ROSC. Subsequent studies have highlighted limitations of coronary perfusion pressure as a determinant of ROSC, in that it does not necessarily reflect coronary blood flow and as aortic diastolic pressures decline over time, results may have been confounded by resuscitation time bias. Nevertheless, the study provided a basis to test drug strategies which focus on the physiological response to their administration.

A piglet model of asphyxia-associated ventricular fibrillation (VF) tested the hypothesis that titrating compression depth to a systolic blood pressure of 90 mmHg and vasopressor administration to maintain coronary perfusion pressure ≥ 20 mmHg improved survival compared to standard advanced life support [35]. The survival rate was greater with haemodynamic-CPR group than standard care (100% v 60%, *P* = 0.03). The study served as the basis for a large multi-centre randomised trial that aimed to evaluate this novel strategy, in combination with performance debriefing, for paediatric cardiac arrest.

The Improving Outcomes from Paediatric Cardiac Arrest—the ICU-Resuscitation Project, was a parallel, hybrid stepped-wedge, cluster randomised trial, involving 18 paediatric intensive care units (ICUs) from 10 North American hospitals [36]. The intervention comprised an ICU resuscitation quality improvement bundle consisting of (1) CPR training at the point of care on a manikin and (2) structured debriefings of cardiac arrest events focusing on physiological outcomes. The trial analysed 1074 paediatric cardiac arrests. More patients in the intervention group than the control group achieved adequate intra-arrest diastolic blood pressure (adjusted OR 2.18 (95% CI 1.04–4.»54)), but this did not translate into improvement in the primary outcome of survival to hospital discharge with favourable neurologic outcomes (53.8% v 52.4%, adjusted OR 1.08 (95% CI 0.76–1.53). There was also no significant difference between groups in ROSC or survival to hospital discharge (58.0% v 56.8%, adjusted OR 1.03 (95% CI 0.73–1.47). The authors speculate that the reasons the intervention was unsuccessful may be related to the study being underpowered due to a higher than anticipated survival rate in the control group. It is also suggested there may have been a ceiling effect from already optimised treatments in the control group and other factors (e.g. underlying illness) may have affected overall patient outcome.

## Vasopressin

Vasopressin, a naturally occurring hormone, given at high doses is a potent vasoconstrictor, acting via V1a receptors located on smooth muscle cells to increase systemic vascular resistance. It was recommended in resuscitation guidelines between 2000 to 2015 [7]. The Cochrane review of vasopressors in cardiac arrest examined the use of vasopressin as an alternative to adrenaline and in addition to adrenaline [7].

### Vasopressin as an alternative to adrenaline

The review identified six randomised controlled trials that compared vasopressin with standard dose adrenaline [7]. Vasopressin improved survival to hospital admission (RR 1.27 (95% CI 1.04–1.54); 3 studies, 1954 participants), but had no effect on ROSC (RR 1.10 (95% CI 0.90–1.33); 6 studies, 2531 participants), survival at hospital discharge (RR 1.25 (95% CI 0.84–1.85); 6 studies, 2511 participants) or favourable neurological outcome (RR 0.82 (95% CI 0.54–1.25); 4 studies, 2406 participants). Evidence certainty was ranked as low for survival to hospital admission and very low for return of spontaneous which may explain, in part, the difference in observed effect despite the outcomes occurring at a similar time-point. The absence of consistent evidence that either drug is superior may be due to their broadly similar pharmacological effects as vasoconstrictors or, possibly, that that previous trials comparing the two drugs were underpowered to detect any small difference in effect.

### Vasopressin in addition to adrenaline

Three randomised controlled trials compared vasopressin and adrenaline with adrenaline alone in out of hospital cardiac arrest [7]. The combination of vasopressin and adrenaline did not improve any short-term or long-term outcome (e.g. survival to hospital discharge RR 0.76 (95% CI 0.47–1.22); three trials, 3242 participants).

### Vasopressin and corticosteroids

The potentially synergistic effects of vasopressin and corticosteroids have been explored in three randomised controlled trials during in-hospital cardiac arrest, published in 2009, 2013 (Greece)[37, 38] and 2021 (Denmark) [39, 40]. The trial interventions comprised 40 mg methylprednisolone and 20 IU vasopressin (up to five doses) in adult in-hospital cardiac arrest patients receiving at least one dose of adrenaline (total sample size 869). Across studies, there was heterogeneity in the characteristics of enrolled patients and post-resuscitation steroid protocols.

A Bayesian, meta-analysis of individual participant data, using non-informative priors as the primary analysis reported the posterior odds ratio for ROSC as 2.13 (95% credible interval 1.51–2.82), whilst effects on long-term outcomes were uncertain (survival to hospital discharge OR 1.39 (95% credible interval 0.81–2.00); favourable neurological outcome 1.65 (95% credible intervals 0.91–2.45) [40]. In analyses with strongly optimistic priors, all outcomes were improved with the intervention whilst in the analyses with strongly pessimistic priors, only ROSC improved with the intervention. The probability of observing a beneficial effect for survival to hospital discharge (i.e. odds ratio > 1.0) ranged from 24% for a strong pessimistic prior to 99% for a strong optimistic prior. Treatment effects were consistent across 6 pre-defined sub-groups (age, time to study drug, witnessed, initial rhythm, aetiology and location). The authors of the review highlight the need for larger trials to determine the effect on long-term outcomes.

## Calcium

Calcium serves as both an inotrope and vasopressor. Until recently, despite a paucity of evidence it has been used relatively frequently in the treatment of patients with cardiac arrest in some systems [41]. The Danish Calcium Out of hospital Cardiac Arrest (COCA) randomised controlled trial examined the addition of calcium (up to two doses of 5 mmol calcium chloride) to standard advanced life support interventions (including adrenaline) amongst 391 adults with out of hospital cardiac arrest [27]. The trial was stopped early after an interim analysis suggested harm in the intervention arm. The final analysis found no difference in the rate of ROSC (19% v 27%, RR 0.72 (95% CI 0.49–1.03)), 30 day survival (5% v 9%, RR 0.57 (95% CI 0.27–1.18)) or favourable neurological outcome (4% v 8%, RR 0.48 (95% CI 0.20–1.12)). The point estimates of the treatment effect for all outcomes were in the direction of harm. These data raise substantial concern about the routine use of calcium as a treatment for cardiac arrest. Whether there remains a role in situations such as cardiac arrest associated with hyperkalaemia requires further research.

## Noradrenaline and phenylephedine

The International Liaison Committee on Resuscitation systematic review and meta-analysis of vasopressors in cardiac arrest identified only two randomised trials of noradrenaline and one which compared adrenaline with phenylephrine [42]. The trials were conducted more than 30 years ago and enrolled relatively low numbers of patients (580 for the noradrenaline trials, 65 the phenylephrine trial). Outcomes were similar between groups. Given the paucity of data, no treatment recommendations were made in relation to these drugs.

## Conclusion

Early recognition of cardiac arrest and calling for help, early CPR and early defibrillation remain the most effective interventions for cardiac arrest. Where initial resuscitation efforts with these interventions are unsuccessful, the use of vasopressors is likely to have a small but important impact on survival. Adrenaline, whilst highly effective at restarting the heart has smaller effects on long-term outcomes (survival and favourable outcomes). To maximise any benefit from adrenaline, it should be given as soon as practicable in patients with non-shockable rhythms.

## Data Availability

Not applicable.
